# Tea consumption and measures of attention and psychomotor speed in the very old: the Newcastle 85+ longitudinal study

**DOI:** 10.1186/s40795-020-00361-8

**Published:** 2020-10-06

**Authors:** Edward Jonathan Okello, Nuno Mendonça, Blossom Stephan, Graciela Muniz-Terrera, Keith Wesnes, Mario Siervo

**Affiliations:** 1 Human Nutrition Research Centre, Population Health Sciences Institute, Newcastle University, Newcastle upon Tyne, NE1 7RU UK; 2 EpiDoC Unit, NOVA Medical School, Universidade Nova de Lisboa (NMS-UNL), Lisbon, Portugal; 3 Comprehensive Health Research Centre (CHRC), Lisbon, Portugal; 4 Institute of Mental Health, School of Medicine, University of Nottingham, Innovation Park, Jubilee Campus, Triumph Road, Nottingham, NG7 2TU UK; 5 Centre for Dementia Prevention, University of Edinburgh, Royal Edinburgh Hospital, Edinburgh, EH16 4UX UK; 6 Wesnes Cognition Ltd, Streatley on Thames, RG8 9RD UK

**Keywords:** Tea, Cognition, Very old, Epidemiology

## Abstract

**Background:**

A number of studies have indicated a beneficial effect of tea consumption on the reduction of risk of cognitive impairment and dementia in older aged populations. However, there is a paucity of data on these associations in the very old, defined as individuals aged 85 years and over. We investigated the relationship between tea consumption in the very old and measures of global cognitive function, memory, attention and psychomotor speed.

**Method:**

Longitudinal (5-years), population-based cohort study of individuals aged 85+ years in the North East of England, United Kingdom. Participants were community-dwelling and institutionalized men and women recruited through general medical practices (*n* = 676). Baseline tea consumption and longitudinal measures of global and domain specific (memory, speed and attention) cognitive function were assessed. Linear mixed models, controlling for demographic (e.g. age, sex and education) and health variables were used to determine whether tea consumption was protective against cognitive decline.

**Results:**

Tea consumption was not associated with cognitive function at baseline on any measure (unadjusted and adjusted analyses). In the linear mixed effects models adjusted for age, sex, education and disease co-morbidity, higher tea consumption was associated with significantly better attention (focused and sustained attention), and psychomotor speed (complex tasks only) over five-years follow-up. However, there was no association between tea consumption and global cognitive function, memory or performance on simple speed tasks over time.

**Conclusions:**

In this cohort study of non-demented very old adults we found that higher (vs. lower) tea consumption was associated with better performance over time on measures of focused and sustained attention and some psychomotor speed tasks. No associations with global cognition, memory or easy speed tasks (simple Reaction Time or Word Recognition) were detected**.** The results have implications for the development of possible diet-based interventions focused on improving cognitive function in the very old age group. These findings need to be confirmed in a sufficiently powered and well-designed RCT with non-demented very old adults.

## Background

The world age demographic is changing rapidly [1]. The World Health Organization (WHO) reports that between 2000 and 2050, the proportion of individuals aged 60 years and over will double from approximately 11 to 22% [2]. The greatest increase will be in persons aged 80 years and older, such that by 2050 the world will have almost 400 million people in this age group [2]. Despite dramatic success in improving human survival, longer life expectancy does not necessarily mean a healthier life [3]. The change in population age structure has been associated with large increases in the prevalence and incidence of age related conditions including cognitive impairment and dementia [4]. In 2013, it was estimated that there were over 46 million people with dementia worldwide and this number is predicted to rise to 131.5 million by 2050 [5]. Rates for severe cognitive impairment (no dementia) are also high and increasing [5]. The public health significance of these trends is dramatic and will impose considerable pressure on individuals, their families and society. The identification of effective strategies to maintain cognitive health with ageing is a research priority to ensure that the ageing transition is successful worldwide.

Several studies have demonstrated that modifiable risk factors such as diet can play a role in maintaining cognitive function and preventing decline in older aged populations [6–8]. Tea, the hot water infusion from the leaves of the plant *Camellia sinensis*, is the most consumed beverage in the world after water. Black tea is the most commonly consumed tea and accounts for 76–78% of world tea consumption [9]. The UK is ranked 3rd in the world for tea consumption per capita after Turkey and Ireland [10]. Tea has been associated with several positive psychosomatic states such as clarity of mind, alertness, enhanced attention and relaxation [11]. To date, no research has been undertaken on the association between tea consumption and cognitive function in the very old population; defined as those aged 85 years and over. Therefore, the aim of this study was to examine the association between tea consumption and cognitive function including measures of global and domain specific (i.e. memory, speed and attention) cognitive performance using data from the Newcastle 85+ longitudinal study. Knowing if tea is protective against cognitive decline in this age group will have implications for the development of intervention strategies focused on improving health and cognitive function.

## Methods

### Participant cohort

**Fig. 1:**
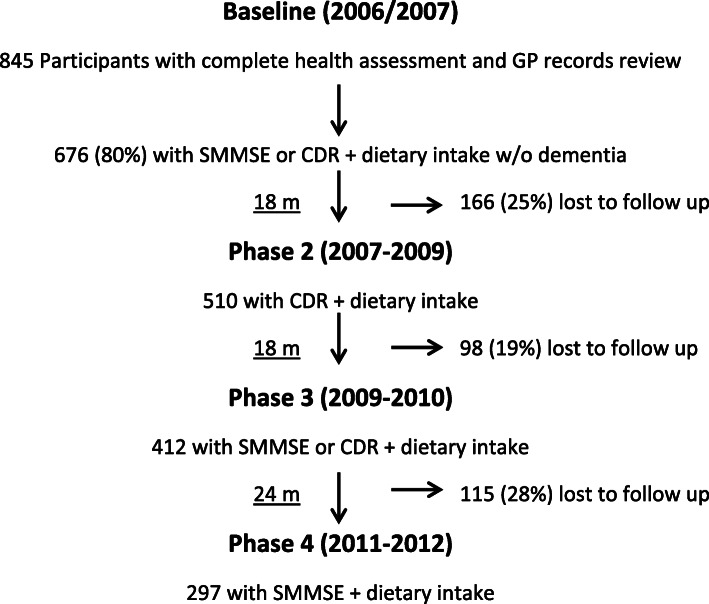
Flowchart of the Newcastle 85+ study participants by SMMSE and CDR scores and dietary intake availability over the study period

The Newcastle 85+ study is a community based longitudinal study on multi-dimensional health and ageing aspects in persons aged 85 years and over in 2006 (born 1921). Participants with end-stage illness; or perceived to pose a risk to the research nurse visiting alone; or were diagnosed with dementia; or without tea consumption or SMMSE/CDR data were excluded from the study. Based on health assessments and General Practice (GP) clinical records, the cohort was broadly representative of the general UK population. Full recruitment, screening, baseline and follow-up repeat health assessment processes and measurements of core variables are described elsewhere [12, 13]. Baseline and follow-up interviews at 18 (1.5 years; wave 2), 36 (3 years; wave 3), and 60 months (5 years; wave 4) were completed by research nurses at the participants’ usual places of residence. Figure 1 indicates the participants’ flow chart for the study.

### Dietary assessment

Tea intake was assessed at baseline by a 24 h Multiple Pass Recall (24 h-MPR) on two non-consecutive occasions administered by trained research nurses. Total daily tea intake was calculated by averaging intakes of both 24 h-MPR. Portion sizes were estimated using the “Photographic Atlas of Food Portion Sizes” [14]. Black tea with or without milk was by far the most widely consumed type of tea (*n* = 738) therefore green (*n* = 3), herbal (*n* = 14) and lemon tea (*n* = 3) (instant powder) were excluded. The tea consumption variable was created by ranking tea intake into tertiles where one cup of tea was assumed to be 200 ml. Two groups were created: high tea consumption (tertile 3. Range 4.56 to 11.85 cups per day; *n* = 463) vs. moderate/low tea consumption (tertiles 1 and 2. Range 0.25 to 4.55 cups per day; *n* = 213).

### Dementia

Diagnosis of dementia was taken from the GP record review.

### Neuropsychological battery

Mini-Mental State Examination (MMSE) was used to assess global cognitive function at baseline, 36 and 60 months [15, 16]. Scores range from 0 to 30, with higher scores indicating better global cognitive performance. Domain specific cognitive performance, including speed, attention and episodic memory were assessed using the Cognitive Drug Research (CDR) computerised system [17–20]. The CDR battery of assessments included scores for: Word Recognition and Recall (WR), Simple Reaction Time (SRT), Digit Vigilance Task (DVT), and Choice Reaction Time (CRT). Five composite measures were derived from the combination of the above scores: Power of Attention (PoA; a measure of focused and time dependent attention), Continuity of Attention (CoA; a combination of and measurement of accuracy of scores from CRT and DVT), response time variability (RTV; a measure of fluxes in attention) and the WR accuracy sensitivity index (SI – an assessment of memory). Lower scores for speed and attention (except CoA) indicate better performance whilst higher scores for SI indicate better performance. The assessment battery took approximately 15 min to complete.

### Covariates

Baseline covariates included in the analysis were: age, sex, years of education and disease co-morbidity (calculated as the sum of the presence or absence of a history of cardiac disease, cerebrovascular disease or diabetes; with scores coded as 0, 1, or 2–3).

### Analysis

Differences in baseline demographic, lifestyle, health and cognitive variables between the high and low tea consumption groups were compared using the χ2 statistic for categorical variables or independent t-test for normally distributed continuous variables (or Wilcoxon Rank sum (Mann-Whitney) test for non-normally distributed variables). We fitted linear mixed effects models to the cognitive measures collected over 3 indicated time points including 18 (1.5 years), 36 (3 years), and 60 months (5 years). Due to the number of time points available for analysis, we estimated models with constant rate of change over time. (i.e. no quadratic term for time was fitted). The intercept was adjusted for baseline age (centred at age 85), sex (0 = male, 1 = female), years of full-time education (centred at 7 years, the average number of years of education in the sample), disease count. Rate of change was adjusted for baseline age, sex, and education. Results are presented as coefficients with 95% confidence intervals (95% CI). An unstructured correlation matrix was considered, which permitted the estimation of residual covariances. Models were estimated using maximum likelihood estimation and estimates were robust under a missing at random assumption. Statistical significance was set a *p* < 0.05. All analyses were completed using Stata Version 14.

## Results

### Demographics

Of the 845 participants who completed the health assessment and GP record review, 738 (87.3%) had information on tea consumption. Of the 738 participants, 676 had no dementia at baseline and form the analytical sample. Table 1 shows the baseline characteristics of the sample by tea status (high vs. low consumption). As shown, there were no significant differences in baseline age, years of education or health status (all conditions and total co-morbidity score) between the low and high tea consumption groups. There was however a gender difference, with a greater number of females in the high (63.9%) compared to the low (54.9%) tea consumption group (*p* = 0.026).

**Table 1:** Baseline characteristics of the study sample (*n* = 676) by tea consumption (high vs. low)

	Low Tea Consumption (***n*** = 213)	High Tea Consumption (***n*** = 463)	***p***-value*
**Demographics**			
Mean age, years (SD)	85.4 (0.4)	85.5 (0.4)	0.099^1^
Mean education, years (SD)	9.8 (1.8)	9.9 (1.8)	0.820^1^
% Female (n)	54.9 (117)	93.9 (296)	0.026
**Health**			
Mean body mass index (SD)	24.5 (4.6)	24.5 (4.2)	0.920^1^
Mean total cholesterol, mmol/l (SD)	4.8 (1.2)	4.8 (1.2)	0.964^1^
Mean waist-to-hip ratio (SD)	0.9 (0.1)	0.9 (0.1)	0.460^1^
% Hypertension (n)	54.7 (116)	62.2 (288)	0.066
% Severe depression (n)	7.2 (15)	8.6 (39)	0.817
% Cardiac disease (n)	46.7 (*n* = 99)	48.0 (*n* = 222)	0.763
% Cerebrovascular disease (n)	17.5 (*n* = 37)	20.3 (*n* = 94)	0.385
% Diabetes (n)	12.7 (*n* = 27)	13.8 (*n* = 64)	0.701
% PVD (n)	6.6 (*n* = 14)	7.6% (*n* = 35)	0.657
% Total disease count ≥2 (n)	20.8 (*n* = 44)	22.5 (*n* = 104)	0.664
**Lifestyle**			
% High physical activity (n)	34.9 (74)	36.8 (170)	0.721
% Current smoking (n)	8.0 (17)	3.5 (16)	0.020
% Heavy alcohol use (n)	7.1 (10)	18.1 (61)	0.008

* Chi-squared test. ^1^ Independent sample t-test

### Baseline cognitive function

There were no significance differences in baseline global or domain specific test scores between the low and high tea consumption groups in the whole sample (shown in Table 1) or in gender stratified analysis (results not shown).

### Longitudinal cognitive performance

Table 2 shows the pattern of associations between tea consumption and cognitive performance on each measure over time when controlling for covariates (for the full model results see Supplementary Appendix 1). Compared to the low tea consumption group people in the high tea consumption group displayed significantly better cognitive function over five-years follow-up on measures of attention (PoA and CoA) and speed (i.e. complex tasks only including choice and digit vigilance RT). The low tea group also had lower performance over time on the response time variability score (i.e. a measure of fluctuations in attention), but this failed to reach statistical significance (*p* = 0.069). Tea consumption was not associated with measures of global cognitive function (i.e. MMSE score), memory or speed on simple tasks (i.e. simple RT or word recognition RT) over 5 years follow-up.

**Table 2:** Baseline cognitive test (median) scores by tea group

	Low Tea Consumption (***n*** = 213)	High Tea Consumption (***n*** = 463)	***p***-value*
**Global Cognitive Function**			
MMSE (IQR)	28 (26, 29)	28 (26, 29)	0.168
**Memory**			
Memory (SI) Index (IQR)	0.61 (0.47, 0.71)	0.60 (0.47, 0.75)	0.462
**Attention**			
Power of Attention IQR)	1.49 (1.35, 1.68)	1.47 (1.35, 1.66)	0.442
Continuity of Attention (IQR)	87.84 (80.67, 91.67)	87.17 (80.84, 100.00)	0.491
Response Time Variability (IQR)	0.06 (0.52, 0.07)	0.59 (0.05, 0.07)	0.675
**Speed**			
Simple RT, sec (IQR)	0.39 (0.34, 0.49)	0.38 (0.34, 0.46)	0.149
Choice RT, sec (IQR)	0.57 (0.52, 0.65)	0.57 (0.52, 0.64)	0.385
Digit Vigilance RT, sec (IQR)	0.52 (0.48, 0.56)	0.51 (0.47, 0.56)	0.886
Word recognition RT, sec (IQR)	1.31 (1.08, 1.85)	1.28 (1.07, 1.70)	0.212

*MMSE* Mini Mental State Examination, *SI* sensitivity index, *RT* reaction time

* Wilcoxon Rank sum (Mann-Whitney) test

## Discussion

In this study, tea consumption was associated with some (e.g. attention and complex speed tasks), but not all (i.e. global, memory and simple speed tasks) cognitive domains over time. The results support previous work of a link between tea consumption and cognition [21–24], extending the findings for the first time to the very-old population and highlight a role of black tea in cognitive performance. The results have important implications for informing the development of possible prevention and risk reduction approaches, of which nutritional interventions are key modifiable factors for age associated cognitive decline or dementia.

Previous studies linking tea consumption to cognitive performance have primarily focused on green tea. The findings have mostly been positive with higher consumption associated with protective effects against global cognitive decline in normal and probable-AD cases and a lower prevalence of cognitive impairment (i.e. MMSE score or comprehensive Global Geriatric Assessment scores [21, 22]. The association has been linked to, inter alia, amount of polyphenol intake and associated lowering of plasma homocysteine, a risk factor for probable dementia. In contrast, a study of community-living Chinese adults in Singapore aged ≥55 years found that regular tea consumption, particularly black and oolong tea (4–7 cups (215 mL/day), was associated with lower risk of cognitive impairment and decline on the MMSE [23]. This was supported by a RCT study of the effects of consumption of black tea and other beverages (400 mL per subject) on cognitive and psychomotor performance which indicated that in the experimental arm where participants drank 400 mL of black tea/day there was a strong association with rapid increases in alertness and information processing capacity [24].

The putative effects of tea consumption on psychological states such as mood and cognitive function are attributed to their phytochemical constituents. Examples of such compounds include, inter alia, the methylxanthine, caffeine which as a central nervous system stimulant has been shown to increase alertness and concentration, notably through its antagonistic effects on adenosine A_1_ and A_2A_ receptors [25]; the amino acid L-theanine which has been shown to have positive effects on mood such as increased calmness and cognitive performance, perhaps through modulation od GABA receptors [26] and various polyphenols with varied positive effects on brain function [11, 27–30]. Studies by Scholey et al. [31] and Okello et al. [32] demonstrated that supplementation with 300 mg of the tea constituent Epigallocatechin Gallate (EGCG) or consumption of either black or green tea increase the Electroencephalogram (EEG) spectral powers of Theta, Alpha and Beta brain wave activities, a potential indication of their putative anxiolytic, attention and concentration effects.

Lifestyle, socio-demographic and health cofounders have a bearing on cognitive function. Although significant differences were observed between the low and high tea consumers in terms of smoking and alcohol use, these differences had no significant effects on the baseline cognitive tests (Tables 1 & 2). Other notable health aspects purported to be associated with cognitive functions are deficiencies in vitamins such as vitamins D and B12. Previous studies based on our 85+ cohort showed no effect of Vitamin D on global cognition over a 3-year observation period although small season-specific incidents of impairment on attention specific tasks were observed [33]. A larger cohort and longer term (12- years) study concluded that there was ‘no association between vitamin D status and long-term risk of dementia or cognitive impairment’ [34]. Another study based on our 85+ cohort showed that neither folate nor plasma vitamin B12 were predictive for cognitive decline over the 5-year study period [35].

## Conclusion

In the very old (85+ years) and non-demented we found that higher (vs. lower) tea consumption was not associated with baseline cognitive performance on any measure. However, higher (vs. lower) tea consumption was associated with better performance over time (5-years) on measures of attention (focused and sustained but not fluctuations – only a trend with these measures) and some speed measures (choice and digit vigilance RT). No association with global cognition, memory or easy speed tasks (simple RT or word recognition RT). These results add to the growing body of research showing that teas derived from the plant *Camellia sinensis*, irrespective of their manufacturing processing and demographic consumption habits have a beneficial effect on cognitive function dependent on frequency of consumption.

### Strengths and limitations

The strengths of the study are large sample size, specific focus on the very old population, and an extensive collection of multidimensional health variables, including longitudinal cognitive follow-up. Limitations include dietary assessments which are based on at baseline on two non-consecutive occasions (e.g. we could not detect if tea consumption declined with time). However tea consumption patterns were stable throughout the week in this population.

## Supplementary information

**Additional file 1.**

## Data Availability

By application to the Data Manager, Newcastle 85+ study Data Guardians Group. http://research.ncl.ac.uk/85plus/

## Abbreviations

CDR: Cognitive Drug Research
CoA: Continuity of Attention
CRT: Choice Reaction Time
DVT: Digit Vigilance Task
EEG: Electroencephalogram
EGCG: Epigallocatechin Gallate
GP: General Practice
MMSE: Mini-Mental State Examination
MPR: Multiple Pass Recall
PoA: Power of Attention
PVD: Peripheral Vascular Disease
RCT: Randomised Controlled Trial
RT: Reaction Time
RTV: Response Time Variability
SI: Sensitivity Index
SRT: Simple Reaction Time
WHO: World Health Organization
WR: Word Recognition
