# Correction to: Tea consumption and measures of attention and psychomotor speed in the very old: the Newcastle 85+ longitudinal study

**DOI:** 10.1186/s40795-021-00409-3

**Published:** 2021-02-08

**Authors:** Edward Jonathan Okello, Nuno Mendonça, Blossom Stephan, Graciela Muniz-Terrera, Keith Wesnes, Mario Siervo

**Affiliations:** 1 Human Nutrition Research Centre, Population Health Sciences Institute, Newcastle University, Newcastle upon Tyne, NE1 7RU UK; 2 EpiDoC Unit, NOVA Medical School, Universidade Nova de Lisboa (NMS-UNL), Lisbon, Portugal; 3 Comprehensive Health Research Centre (CHRC), Lisbon, Portugal; 4 Institute of Mental Health, School of Medicine, University of Nottingham, Innovation Park, Jubilee Campus, Triumph Road, Nottingham, NG7 2TU UK; 5 Centre for Dementia Prevention, University of Edinburgh, Royal Edinburgh Hospital, Edinburgh, EH16 4UX UK; 6 Wesnes Cognition Ltd, Streatley on Thames, RG8 9RD UK

**Correction to: BMC Nutr (2020) 6:57**

**https://doi.org/10.1186/s40795-020-00361-8**

Following publication of the original article [1], the authors reported that Table 3 was mistakenly omitted from the published article. Table 3 is supplied below. In the Results section of the original article, the following changes are made: Baseline cognitive function: "(shown in Table 1)" should read '(shown in Table 2)'. Longitudinal cognitive performances: 'Table 2 shows..' should read 'Table 3 shows..'.

**Table 3:** Results of the mixed multilevel analyses of the effect of tea consumption on performance on the MMSE and CDR memory, attention and speed scores over 5 years follow-up

	Coefficient*	95%CI	***p***-value
**Global Cognitive Function**			
MMSE	0.14	(-0.10 to 0.38)	0.247
**Memory**			
Memory (SI) Index	0.01	(0.00 to 0.03)	0.144
**Attention**			
Power of Attention	**-0.04**	**(-0.08 to -0.01)**	**0.017**
Continuity of Attention	**1.30**	**(0.62 to 1.99)**	**<0.001**
Response Time Variability	0.00	(0.00 to 0.00)	0.069
**Speed**			
Simple RT	-0.01	(-0.03 to 0.01)	0.176
Choice RT	**-0.02**	**(-0.04 to 0.00)**	**0.042**
Digit Vigilance RT	**0.00**	**(-0.01 to 0.00)**	**0.017**
Word Recognition RT	-0.03	(-0.09 to 0.04)	0.418

* All models were controlled for age, sex, years of full time education, age*time, sex*time, education*time and baseline disease co-morbidity score. *MMSE* Mini Mental State Examination, *SI* sensitivity index, *RT* reaction time
